# Mucosal-Associated Invariant T Cells in T-Cell Non-Hodgkin Lymphomas: A Case Series

**DOI:** 10.3390/cancers14122921

**Published:** 2022-06-14

**Authors:** Pietro Torre, Annalisa Brescia, Giorgio Giurato, Raffaella D’Auria, Francesca Rizzo, Benedetta Maria Motta, Valentina Giudice, Carmine Selleri, Pio Zeppa, Alessandro Caputo, Vincenzo Casolaro, Marcello Persico

**Affiliations:** 1 Internal Medicine and Hepatology Unit, University Hospital “San Giovanni di Dio e Ruggi d’Aragona”, University of Salerno, 84131 Salerno, Italy; ptorre@unisa.it; 2 Department of Medicine, Surgery and Dentistry, “Scuola Medica Salernitana”, University of Salerno, 84081 Baronissi, Italy; annalisa.brescia@gmail.com (A.B.); ggiurato@unisa.it (G.G.); radauria@unisa.it (R.D.); frizzo@unisa.it (F.R.); bmotta@unisa.it (B.M.M.); vcasolaro@unisa.it (V.C.); 3 Hematology and Transplant Center, University Hospital “San Giovanni di Dio e Ruggi d’Aragona”, University of Salerno, 84131 Salerno, Italy; vgiudice@unisa.it (V.G.); cselleri@unisa.it (C.S.); 4 Pathology Unit, University Hospital “San Giovanni di Dio e Ruggi d’Aragona”, University of Salerno, 84131 Salerno, Italy; pzeppa@unisa.it (P.Z.); alessandro.caputo@unina.it (A.C.)

**Keywords:** MAIT cells, MR1-Ag tetramer, unconventional lymphocytes, T-cell lymphoma

## Abstract

**Simple Summary:**

Mucosal-associated invariant T (MAIT) cells are a subgroup of T lymphocytes whose role has recently been investigated in several types of diseases, including cancer. However, little is known about these cells in lymphomas. In this case series, we investigated the presence of MAIT cells in biopsies obtained from patients diagnosed with T-cell non-Hodgkin lymphomas, uncommon hematological malignancies with often not clearly defined etiopathology.

**Abstract:**

Background: Mucosal-associated invariant T (MAIT) cells are a subset of unconventional T lymphocytes expressing a semi-invariant α/β T-cell receptor (TCR). The physiological functions of these cells, which are particularly abundant in normal liver and mucosal sites, have become clear only in recent years, but their role in most human diseases is still unknown. Since the cellular origin and etiopathogenesis of most T-lymphomas are still elusive, we decided to explore the presence of MAIT cells in biopsies from these neoplasms. Methods: Sixteen biopsies obtained from patients with a T-cell lymphoma diagnosis were analyzed via immunofluorescence staining using an anti-Vα7.2 antibody and the MR1-antigen tetramer. Positive cases were subjected to a polymerase chain reaction for the detection of Vα7.2–Jα33, Vα7.2–Jα20, or Vα7.2–Jα12 rearrangements, followed by sequencing of the CDR3α region. Results: CD3+/Vα7.2+ and CD3+/MR1-Ag-tetramer+ cells were found in 4 of 16 samples analyzed. The identification of specific TCR rearrangements confirmed the presence of these cells in all four samples. PCR and sequencing results documented the presence of multiple clones of MAIT cells in each positive sample. Conclusions: MAIT cells are frequently found in T-cell lymphomas. More in-depth studies and a larger number of samples are needed to better clarify the contribution of MAIT cells to this rare neoplasm.

## 1. Introduction

**Figure 1:**
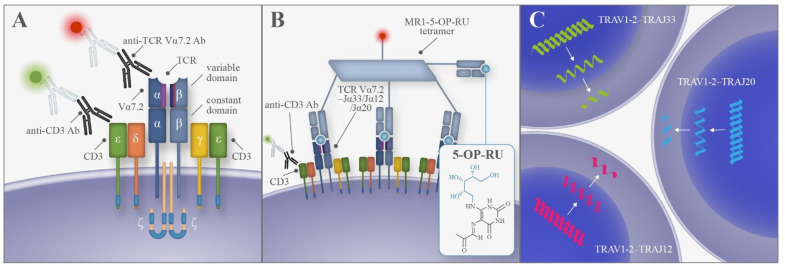
T-cell receptor (TCR) restriction in MAIT cells and their identification: (**A**) MAIT cells are T lymphocytes (CD3^+^ cells) that express a semi-invariant TCR including the Vα7.2 segment associated with a limited selection of Jα segments (Jα33, Jα12, or Jα20). Available antibodies can individually recognize the Vα7.2 segment and CD3; (**B**) TCR of MAIT cells can bind non-protein antigens, such as the potent activator 5-(2-oxopropylideneamino)-6-D-ribitylaminouracil (5-OP-RU), mounted on non-polymorphic MHC class I-related (MR1) protein; (**C**) TCR VαJα rearrangements in MAIT cells.

Mucosal-associated invariant T (MAIT) cells constitute an abundant subset of atypical T lymphocytes whose distinctive features have only been described in recent years. MAIT cells and other subtypes of T lymphocytes, such as natural killer T (NKT) cells, γδ-T cells, and germline-encoded mycolyl lipid-reactive (GEM) T cells, have been collectively termed “unconventional” or “innate-like” lymphocytes because they do not recognize classical antigens and do not display biological features that enable their definition as strictly belonging to the innate or adaptive immune system [1,2,3]. In humans, MAIT cells express a semi-invariant αβ T-cell receptor (TCR) more frequently composed of a Vα7.2 (TRAV1-2)–Jα33 (TRAJ33) α-chain, or less frequently a Vα7.2–Jα12 (TRAJ12) or Vα7.2–Jα20 (TRAJ20) α-chain. These poorly variable α-chains are then paired with a limited repertoire of β-chains [1,2,3,4,5]. MAIT cells recognize antigens on non-polymorphic MHC class I-related (MR1) proteins. The most studied and potent activator is 5-(2-oxopropylideneamino)-6-D-ribitylaminouracil (5-OP-RU), a metabolite derived from bacterial or fungal vitamin B2 (riboflavin) biosynthesis [1,2,3,4,5,6]; however, several non-microbial substances can also bind to MR1, expanding the range of possible antigens that MAIT cells can recognize. Moreover, cytokines can activate MAIT cells in a TCR-independent manner and activated cells can rapidly produce pro-inflammatory cytokines and release cytotoxic substances [2,3,6,7]. MAIT cells represent 1–10% of circulating T lymphocytes, but they reach much higher percentages in some organs, such as the liver (up to 45% of total T cells) and the intestine, where they preferentially migrate because of their homing receptor panel. Their frequency was found to be different in patients affected by inflammatory/autoimmune disease, infections, cancer, and as a function of age [1,2,3,8]. Although MAIT cell phenotype can change under specific conditions, these lymphocytes are usually double-negative for CD4 and CD8 (or less frequently CD8^+^, CD4^+^, or double-positive), CD3^+^Vα7.2^+^CD161^hi^IL-18Rα^+^CD26^+^CD45RO^+^ and express the promyelocytic leukemia zinc finger (PLZF) transcription factor [2,3,6]. Some of these markers have been used in variable combinations to identify MAIT cells, but most consistently, these lymphocytes are defined as MR1-antigen-restricted cells, which express a TCR with the aforementioned features (Figure 1) [2,6].

Neoplasms of T-cell origin account for up to 15% of non-Hodgkin lymphomas (NHLs) and have a worse prognosis compared with B-cell malignancies, because of the lack of specific targeted therapies; cellular origin and etiopathogenesis are still open questions likely because most T-cell lymphomas are rare or because of their great heterogeneity [9,10,11,12]. Both conventional T lymphocytes and innate-like T cells have shown that they can undergo malignant transformation [13,14,15]. Following earlier observations of MAIT cells presence in a hepatosplenic αβ T-cell lymphoma (our unpublished results), and in two peripheral T-cell lymphomas not otherwise specified (PTCL-NOS) [14], we investigated the presence and characteristics of MAIT cells in a case series of 16 patients diagnosed with T-cell NHLs via immunofluorescence staining, as well as polymerase chain reaction (PCR) to detect specific semi-invariant TCR rearrangements, and next-generation sequencing to study the complementarity determining region 3 (CDR3) of the TCRα chain in the positive cases.

## 2. Patients and Methods

### 2.1. Patients and Chemotherapy Regimens

For investigation of the presence of MAIT cells in T-cell NHLs, a total of 21 biopsies performed at diagnosis in the period 2014–2019 from patients with T-cell NHLs were retrospectively selected from the archive of the Anatomic Pathology Department, University of Salerno, University Hospital “San Giovanni di Dio e Ruggi d’Aragona”, Salerno, Italy. The corresponding formalin-fixed paraffin-embedded tissue blocks were retrieved, and additional microtome sections were cut. The opening of this study was notified to the ethics committee "Comitato Etico Campania Sud". The study was conducted in accordance with the Declaration of Helsinki [16] and after informed consent was obtained. Of these 21 samples, 5 were not further processed due to the poor quality of the starting material, and tests were conducted on the remaining 16 biopsies. A total of 10 males (62.5%) and 6 females (37.5%) were included, and the median age at diagnosis was 60 years old (range, 17–79 years) (Table 1).

Patients were diagnosed with T-cell neoplasms based on 2008 and 2016 World Health Organization (WHO) criteria [17,18], and 11 of them received chemotherapy as per standard international guidelines at the Hematology and Transplant Center, University Hospital “San Giovanni di Dio e Ruggi d’Aragona”, Salerno, Italy, while 2 subjects died before starting any treatment. Clinical data and outcomes were not available for three subjects. Specifically, seven subjects received CHOEP/CHOP as first-line treatment (doxorubicin, vincristine, cyclophosphamide, and prednisolone with or without etoposide), and one subject with nasopharyngeal localization received dexamethasone, methotrexate, iFOSFamide, asparaginase, and etoposide (SMILE protocol). Two patients with T-ALL cortical types were enrolled in the Gruppo Italiano Malattie EMatologiche dell’Adulto (GIMEMA) LAL1913 protocol and received an autologous hematopoietic stem-cell transplantation (auto-HSCT) after achieving a complete remission post-first-line treatment [19]. One subject received low-dose cyclophosphamide as palliative treatment. Second-line treatments were gemcitabine, oxaliplatin, and dexamethasone (GDP protocol); cyclophosphamide, asparaginase, vincristine, etoposide, and prednisone (COEPL); bendamustine, gemcitabine, and vinorelbine (BeGEV protocol); or brentuximab-vedotin, followed by radiation therapy and low-dose cyclophosphamide. Two ALCL (one ALK+ and ALK-) patients received an auto-HSCT after first-line treatment, while one PTCL-NOS after a second-line therapy because of partial remission after CHOEP.

### 2.2. Immunofluorescence Staining for MAIT Cells Identification

Sections were processed for analysis of cells expressing CD3 (1:50, anti-CD3ε, ab5690, Abcam), Vα7.2 (1:10, 3C10, BioLegend, San Diego, CA, USA), and binding the hMR1 5-OP-RU-PE tetramer (1:50, NIH Tetramer Core Facility, Emory University) [20]. After deparaffinization and rehydration, sections were treated for antigen retrieval with 10 mM citrate buffer (pH 6.0) and heated (90 °C) for 40 min. Slides were cooled for 30 min in antigen retrieval buffers at room temperature, then washed three times with PBS for 10 min. Sections were incubated overnight with the primary antibody and/or 5-OP-RU tetramer and then for 1 h with fluorescent-dye-conjugated secondary antibodies. All images were acquired and analyzed with an inverted TCS SP5 Leica laser-scanning confocal microscope, using a 40x magnification objective (Leica Microsystems), as previously described [21].

### 2.3. DNA Extraction, Amplification, and Purification for TCR VαJα Rearrangements Detection via Deep Sequencing

For TCR VαJα rearrangements detection, three 10 µm sections from each biopsy were collected in a microfuge tube and DNA was extracted using the AllPrep DNA/RNA FFPE Kit (QIAGEN, Hilden, Germany) according to the manufacturer’s instructions. Briefly, 12.5 ng of DNA was amplified, in a total reaction volume of 25 μL, with PCR using KAPA HiFi HotStart ReadyMix (Roche) and 0.2 μM of the following primers, Vα7.2–Jα33: forward (FW) 5′-AGTCGGTCTAAAGGGTACAGGTT-3′, reverse (RW) 5′-TCCCAGCGCCCCAGATTA-3′; Vα7.2–Jα20: FW 5′-AGTCGGTCTAAAGGGTACAGGTT-3′, RW 5′-CAGTTACTGTGGTTCCGGCT-3′; Vα7.2–Jα12: FW 5′-AGTCGGTCTAAAGGGTACAGGTT-3′, RW 5′-CCCAGCGCCCCAGATTAAC-3′. Since PCR had been performed for sequencing library preparation on an Illumina platform, primers were designed with a specific overhang sequence, as reported below:

FW overhang: 5′-TCGTCGGCAGCGTCAGATGTGTATAAGAGACAG-[locus- specific sequence];

RW overhang: 5′-GTCTCGTGGGCTCGGAGATGTGTATAAGAGACAG-[locus- specific sequence].

PCR products were detected via electrophoresis in 2% agarose gels (Sigma-Aldrich, St. Louis, MI, USA) and SYBR safe staining; then, bands were cut for the gel and purified using QIAquick Gel Extraction Kit (QIAGEN, Hilden, Germany), and the obtained samples were used for library preparation.

Afterward, 20 ng of each amplicon was used to prepare the sequencing libraries. Dual indices and Illumina sequencing adapters were attached using a Nextera XT Index Kit (illumine) and KAPA HiFi HotStart ReadyMix (Roche) with PCR using the following program: 98 °C for 3 min; 98 °C for 30 sec, 55 °C for 30 sec, 7 °C for 45 s for 15 cycles; final extension: 72 °C for 5 min. The generated libraries were purified with Agencourt AMPure XP (Beckman-Coulter, Brea, CA, USA), quantified with Qubit fluorometer with the dsDNA HS (high sensitivity) Assay Kit (Thermo Fisher Scientific, Waltham, MA, USA); size was verified by TapeStation (Agilent, Santa Clara, CA, USA) and finally, libraries were diluted to 4 nM. The libraries were pooled and combined with equimolar PhiX (50% total volume) to artificially increase the genetic diversity. DNA sequences of 2 × 75 bp were generated with a MiSeq instrument (Illumina, San Diego, CA, USA), loading the pool at 10 pM final concentration. About 1.5 M reads per sample were obtained.

### 2.4. Data Analysis

Quality control of sequence reads was performed using FASTQC (https://www.bioinformatics.babraham.ac.uk/projects/fastqc/ (accessed on 18 April 2022). Adapter sequences and low-quality reads were removed using Trimmomatic v0.39 set with default parameters [22]. Clonotype identification and quantification were performed using MiXCR v2.1.10 [23], with default parameters. Only clonotypes supported by at least 10 sequences were considered for further analysis. Logos were generated using WebLogo v2.8.2 [24].

## 3. Results

### 3.1. MAIT Cells Identification

**Figure 2:**
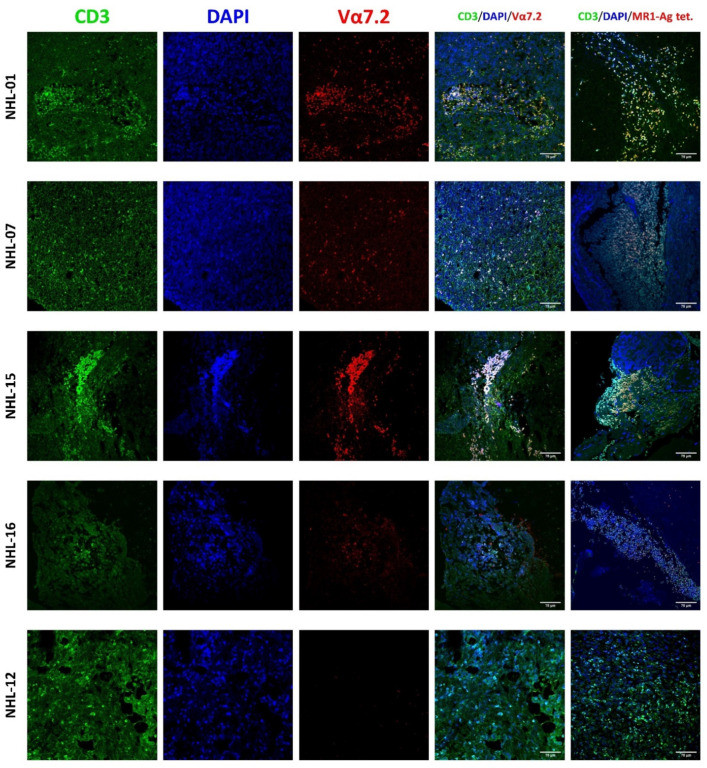
Immunofluorescence staining for MAIT cells detection. Tissues were stained for CD3 (green signal), Vα7.2, or MR1-Ag tetramer (red signal), and nuclei were stained using 4′,6-diamidino-2-phenylindole (DAPI; blue signal). Immunofluorescence staining showed colocalization of CD3/Vα7.2 or MR1-Ag tetramer in biopsies of NHL-01, NHL-07, NHL-15, and NHL-16. NHL-12 is displayed as a negative case.

**Figure 3:**
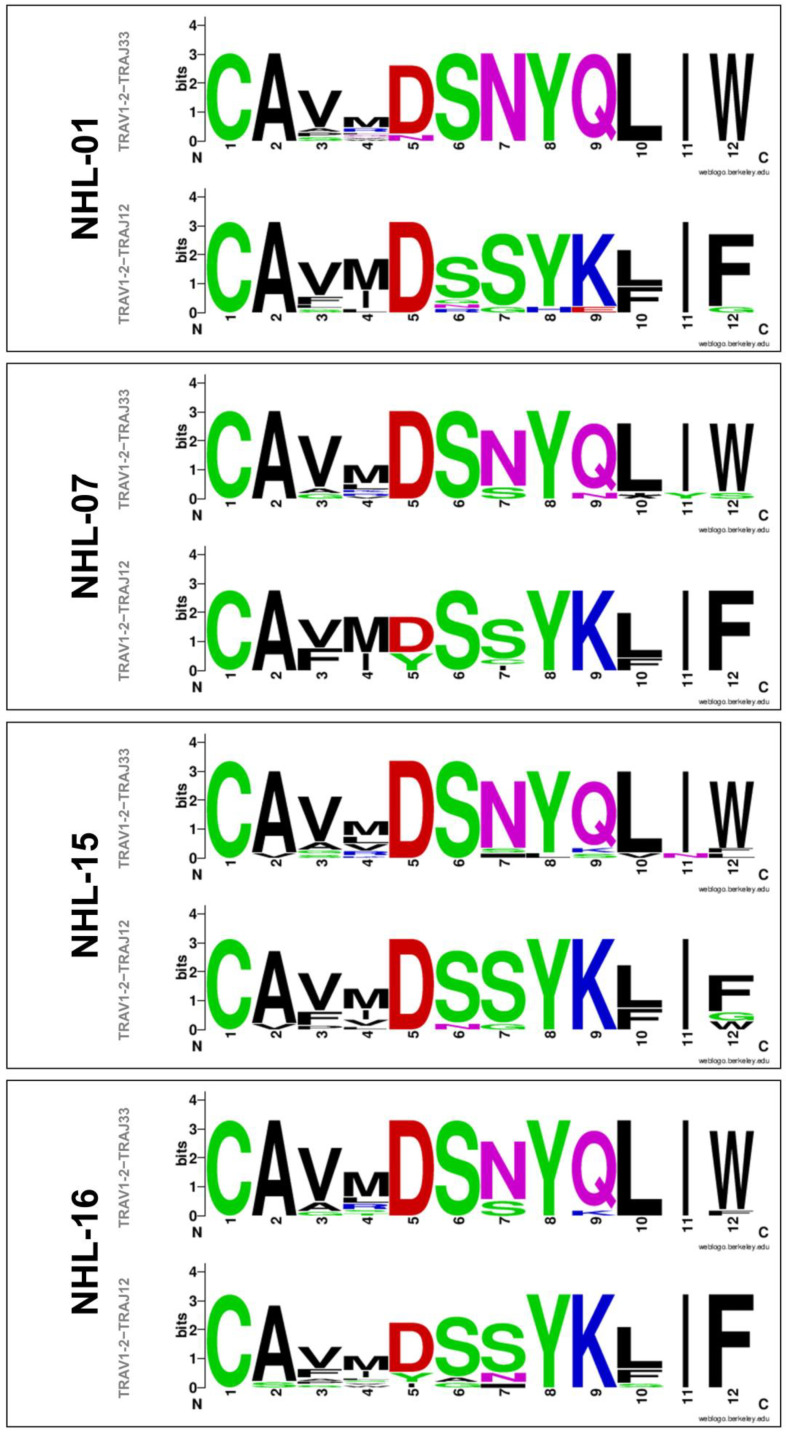
Amino acid sequence logos corresponding to the CDR3α region resulting from MAIT cells TRAV1-2−TRAJ33 or TRAV1-2−TRAJ12 rearranged genes found in T-lymphoma biopsies. Amino acids (represented by letters of various heights) in the different positions are shown on the *x*-axis. The height of each letter in a column is proportional to the amino acid frequency in that position. The total height of the column represents the conservation of the sequence in that position (measured in bits, *y*-axis).

To investigate the presence of MAIT cells in T-lymphoma biopsies, immunofluorescence staining using an anti-Vα7.2-specific antibody and the MR1-5-OP-RU tetramer was performed and analyzed via confocal microscopy (Figure 2). CD3 colocalized with Vα7.2 and MR1-Ag tetramer in four samples; the ratio of Vα7.2^+^/CD3^+^ cells or MR1-Ag-tetramer^+^/CD3^+^ cells on the total of CD3^+^ cells was variable, meaning that MAIT cells were present in different numbers in the analyzed samples. In contrast, CD3^+^ cells did not show a simultaneous positivity for Vα7.2 or MR1-5-OP-RU tetramer in the analyzed fields of the remaining other samples (Table 2 and Figure 2). To confirm the presence of MAIT cells in the positive cases, biopsies were investigated for the presence of semi-invariant TCRs spanning the TRAV1-2−TRAJ33, TRAV1-2−TRAJ12, or TRAV1-2−TRAJ20 rearrangements. MAIT cells-specific TCR rearrangements were found in each of the four samples, but TRAV1-2−TRAJ20 sequences were expressed at lower levels than the established cutoff and were not further analyzed. Figure 3 provides a graphical representation of the amino acid composition of the CDR3α loop resulting from TRAV1-2−TRAJ33 and TRAV1-2−TRAJ12 rearranged genes, showing the relative frequency of different amino acids in each position. In all four analyzed biopsies, there was some variability in the CDR3α region for each rearrangement, particularly in the junction region, suggesting that MAIT cells with different TCRs, and therefore multiple different clones, were present in each T-lymphoma biopsy.

### 3.2. Clinical Characteristics and Outcomes of Patients with Positive Biopsy to MAIT Cells

The presence of MAIT cells was documented in biopsies of 4/16 patients (25%; 95% CI, 9.7–50%). The median age at diagnosis of those subjects was 60 years old (range, 55–72), and three of them were males and one female. MAIT cells were found in biopsies of patients with a previous diagnosis of PTCL-NOS, T-/NK-cell nasal type NHL, or ALK-negative ALCL. In particular, this latter patient (NHL-07, male, 63 years old) had a history of kidney transplants and was admitted to the emergency room for an event of paraplegia. After detection of multiple localizations to the spinal cord and vertebrae, the patient underwent vertebral biopsy, and he died after three weeks due to post-surgery complications. Of the two patients diagnosed with nasal T-/NK-cell type NHL, one (NHL-16, male, aged 72 years old) was first admitted to the Otorhinolaryngology Unit where he received nasal and oral cavity biopsy, but he was then lost at follow-up. The other patient (NHL-15, male, 57 years old), after diagnosis, started the first cycle of chemotherapy as per SMILE protocol, but this was interrupted because of acute renal failure. He was then switched to the COEPL protocol consolidated with radiotherapy. The patient achieved a complete remission (CR), and he is still alive and in good condition at the time of writing. The last patient (NHL-01, female, 55 years old) received a diagnosis of PTCL-NOS in March 2014 and was treated with six cycles of chemotherapy as per CHOEP protocol, showing a negative PET-CT scan with the presence of 0.14% of leukemic CD3^+^CD5^+^CD4^+^CD7^-^CD8^-^ T cells at the bone marrow (BM) aspirate. For this reason, she was treated with GDP as second-line therapy, followed by autologous HSCT (September 2014) after conditioning with thiotepa, cytarabine, etoposide, and melphalan. She is still in CR and in good condition at the time of writing.

Regarding histological features, we observed that, in our series, MAIT cells were frequently found in extranodal sites, particularly in the nasal–oral cavity. No patient with positive biopsy to MAIT cells was diagnosed with an intestinal T lymphoma or a T-cell ALL. Table 3 shows the characteristics of patients with or without MAIT-cell-positive biopsy. Given the small sample size and the lack of follow-up data for all patients, we refrain from providing detailed information on overall survival (OS) and progression-free survival (PFS), but we only report that a reduced PFS was observed in our series for patients with MAIT-cell-positive biopsies (4.7 months vs. 23.9 months) and similar OS between the two groups.

## 4. Discussion

T-cell NHL is a rare condition and histological and phenotypical definition is still challenging because of the lack of knowledge of their deep biological mechanisms [9,10]. In fact, the cellular origin of most T-cell NHL subtypes is still poorly defined, while increasing knowledge might provide useful clues on pathogenesis and potential therapeutic targets. In recent years, novel unconventional T-cell subsets have been studied in several human diseases, including autoimmune disorders and cancers [1,2,3]. PLZF is a master transcriptional factor of NKT cells [25] and was found frequently expressed in lymphoid malignancies such as T-cell ALL, but also in PTCL-NOS and mycosis fungoides biopsies [15]. NKT cells are characterized by CD1d restriction and expression of invariant TCRα chain (Vα24–Jα18) and can be identified using a CD1d tetramer loaded with a synthetic α-galactosylceramide (αGalCer) antigen [26,27]. The absence of the TCR Vα24 in PLZF positive PTCL-NOS and mycosis fungoides biopsies led to the conclusion that some lymphomas might originate from innate T lymphocytes different from NKT cells [15]. Later in 2014, MAIT cell origins of two PTCL-NOS based on the simultaneous PLZF expression and the presence of TRAV1-2–TRAJ33 rearrangement were claimed [14]. However, the clonality of MAIT cells TCRs was not described in the positive cases found in that study. The existence of “MAIT cell lymphomas” is an intriguing hypothesis, because could it define a new subset of T-cell malignancy and could suggest new therapeutic approaches. In fact, one might think of therapeutically targeting MAIT-cells-related proteins, such as ABCB1, or the interaction between their TCR and the MR1 protein [2,14]. This cell subtype is not routinely investigated in clinical diagnostics, and a univocal and easy method for identification of MAIT cells in biological fluids and tissues is required to investigate the presence and frequency of MAIT cells in a reliable and reproducible manner [2,6]. The most used combination, Vα7.2/CD161, proved not to be accurate, especially in certain conditions such as cell activation, because activated MAIT cells were found negative for CD161, and conventional T cells carrying a Vα7.2 are constitutively negative for this marker. Immature MAIT cells are also negative for CD161. In addition, other unconventional T lymphocytes (GEM T cells) express the Vα7.2 segment [2,3,4,6,28,29,30,31]. Similarly, PLZF expression was found reduced in MAIT cells upon stimulation, thus making this marker unsuitable for identification of MAIT cells in chronic activation [32]. MAIT cells recognize antigens on MR1 protein, and 5-OP-RU is a potent activator [6,7]. Therefore, the MR1-5-OP-RU tetramer can specifically identify MAIT cells [2,20,33]. To increase the specificity and sensitivity of MAIT cells identification, in our experiments, we combined Vα7.2 and MR1-Ag tetramer staining, as TRAV1-2-negative/MR1-Ag-positive cell subsets were also described [2,6,34,35].

In this case series, we retrospectively investigated the presence of MAIT cells in biopsies of patients diagnosed with T-cell lymphoma and documented their presence in four biopsies based on the combination of the results of immunofluorescence staining and TCR analysis. PCR and sequencing data demonstrated the presence of MAIT cells harboring different Jα segments in each sample, and some variability was found in the CDR3α region even in the same cell “subtype”. CDR3 is the region of the TCR chains responsible for antigen recognition and hosting the greatest variability, as the result of the V(D)J recombination events that occur during T-cell intrathymic maturation. TCR analysis is a widely used method to assess clonality in lymphoproliferative diseases, and for diagnostic purposes, the TCR γ gene is usually analyzed [36,37,38]. During maturation TCR δ, TCR γ, TCR β, and TCR α genes are consecutively rearranged [37,39,40]. In our case series, we used primers for selected Vα and Jα genes to confirm the presence of MAIT cells in the analyzed samples and, once confirmed, to include and study the CDR3α region. The finding of monoclonality (lymphocytes with identical TCRs, also in the CDR3 region) confirms the diagnosis of T-cell malignancy in an appropriate clinical and pathological setting, but the presence of a prevalent clone of T lymphocytes has also been described in non-neoplastic diseases [36,41]. Furthermore, monoclonality was found in some lymphoma biopsies when analyzing the TCR γ gene, but the same samples were found to be multiclonal for the TCR α or β genes, suggesting that if oncogenic transformation is due to a mutation causing cell proliferation before completion of the rearrangement of all TCR loci, one could observe monoclonality of the genes that rearrange first and multiple clones for the genes that rearrange later [41,42].

To our knowledge, this is the first study that analyzes clonality of MAIT cells based on the sequence of the CDR3α region in biopsies of T-cell lymphomas, and this was the first time that such cells were identified in lymphomas other than PTCL-NOS. We found that MAIT cells are frequently present in T-cell lymphomas, suggesting a possible biological role in their pathophysiology, although the precise contribution to the disease, possibly different from case to case, has not been elucidated. However, our study could pave the way for further research in this area. Important limitations of this study include the low initial quality of histological specimens due to their extensive use for diagnostic purposes, the small number of biopsies analyzed and patients included, and the unavailability of follow-up data for all patients, which make it difficult to generalize our observations and fully understand their clinical implications.

## 5. Conclusions

Better biological and clinical knowledge of T-cell NHLs is needed to improve the treatment and outcome of those patients. In our case series, we added evidence of the possible involvement of MAIT cells in T lymphomas by finding that these cells are frequently present in biopsies of such neoplasms. Our preliminary results require further validation in larger prospective studies and the biological role of MAIT cells in T lymphomas should be better investigated by additional in vitro studies.

## Figures and Tables

**Table 1:** Patient characteristics.

Characteristics	*n* = 16
Age, median (range)—years	60 (17–79)
M/F	10/6
Diagnosis	
PTCL—NOS	5
ENKTL-NT	2
EATL	2
Intestinal TCL	1
ALCL	
ALK+	2
ALK−	2
T-ALL	2
Extranodal sites	
Bone marrow (BM)	4
Skin	2
Gastrointestinal	3
Central nervous system (CNS)	1
Nasopharyngeal	2
First-line treatment	11/16
CHOEP/CHOP	7
SMILE	1
LAL1913	2
Cyclophosphamide	1
Autologous HSCT	5/11
Refractory/relapse	7/11
Second-line treatment	
GDP	3
COEPL	1
BeGEV	1
Bendamustine	1
Brentuximab-vedotin/cyclophosphamide	1

Abbreviations. PTCL-NOS, peripheral T-cell lymphoma not otherwise specified; ENKTL-NT, extranodal NK-/T-cell lymphoma, nasal type; EATL, enteropathy-associated T-cell lymphoma; TCL, T-cell lymphoma; ALCL, anaplastic large-cell lymphoma; T-ALL, T-cell acute lymphoblastic leukemia; HSCT, hematopoietic stem-cell transplantation. Protocols’ abbreviations are reported in the text.

**Table 2:** Results of immunofluorescence and PCR, followed by DNA sequencing in our case series.

UPN	Diagnosis	Tissue	CD3	Vα7.2	MR1-Ag tet.	MAIT Cell TCRs
NHL-01	PTCL-NOS	Lymph node	+	+	+	+
NHL-02	PTCL-NOS	Lymph node	+	-	-	/
NHL-03	PTCL-NOS	Lymph node	+	-	-	/
NHL-04	PTCL-NOS	Lymph node	+	-	-	/
NHL-05	PTCL-NOS	Skin	+	-	-	/
NHL-06	ALCL Alk-	Lymph node	+	-	-	/
NHL-07	ALCL Alk-	Spinal cord/vertebrae	+	+	+	+
NHL-08	ALCL Alk+	Lymph node	+	-	-	/
NHL-09	ALCL Alk+	Lymph node	+	-	-	/
NHL-10	EATL	Ileum	+	-	-	/
NHL-11	EATL	Ileum	+	-	-	/
NHL-12	Intestinal TCL	Ileum	+	-	-	/
NHL-13	T-ALL	Lymph node	+	-	-	/
NHL-14	T-ALL	Lymph node	+	-	-	/
NHL-15	ENKTL-NT	Nasal cavity	+	+	+	+
NHL-16	ENKTL-NT	Nasal/oral cavity	+	+	+	+

Abbreviations. PTCL-NOS, peripheral T-cell lymphoma not otherwise specified; ALCL, anaplastic large-cell lymphoma; EATL, enteropathy-associated T-cell lymphoma; TCL, T-cell lymphoma; T-ALL, T-cell acute lymphoblastic leukemia; ENKTL-NT, extranodal NK-/T-cell lymphoma, nasal type.

**Table 3:** Characteristics of patients included in this study.

Characteristics	MAIT Cells+ (*n* = 4)	MAIT Cells− (*n* = 12)
Age, median (range)—years	60 (55–72)	58 (17–79)
M/F	3/1	7/5
Diagnosis		
PTCL—NOS	1	4
ENKTL-NT	2	-
EATL	-	2
intestinal TCL	-	1
ALCL	1	3
T-ALL	-	2
Extranodal sites	4/4	7/12
Bone marrow (BM)	1	3
Skin	-	2
Gastrointestinal	-	3
Central nervous system (CNS)	1	-
Nasopharyngeal	2	-
PFS	4.7 months	23.9 months
3 years OS	66.7%	50%

Abbreviations: PTCL-NOS, peripheral T-cell lymphoma not otherwise specified; ENKTL-NT, extranodal NK-/T-cell lymphoma, nasal type; EATL, enteropathy-associated T-cell lymphoma; TCL, T-cell lymphoma; ALCL, anaplastic large-cell lymphoma; T-ALL, T-cell acute lymphoblastic leukemia. PFS, progression-free survival; OS, overall survival.

## Data Availability

Additional data are available upon request by the authors.
